# Perspective: Implications of Docosahexaenoic Acid and Eicosapentaenoic Acid Supplementation on the Immune System during Cancer Chemotherapy: Perspectives from Current Clinical Evidence

**DOI:** 10.1016/j.advnut.2025.100464

**Published:** 2025-06-14

**Authors:** Jaqueline Munhoz, Vera Mazurak, Catherine J Field

**Affiliations:** Department of Agricultural Food and Nutritional Science, Faculty of Agricultural, Life and Environmental Sciences, 4-126 Li Ka Shing Center for Health Research Innovation, University of Alberta, Edmonton, AB, Canada

**Keywords:** omega-3 fatty acids, inflammation, immune function, chemotherapy, dietary supplements, docosahexaenoic acid, eicosapentaenoic acid

## Abstract

Docosahexaenoic acid (DHA) and eicosapentaenoic acid (EPA) are omega-3 long-chain polyunsaturated fatty acids (n-3 LCPUFAs) with pleiotropic effects on the immune system. Although several preclinical studies support their potential to enhance cancer treatment efficacy, this has not yet been translated into clinical studies. Currently, there are no official recommendations for n-3 LCPUFAs supplementation during cancer chemotherapy. This review examined human studies that supplemented DHA and/or EPA in patients with cancer undergoing chemotherapy, aiming to evaluate n-3 LCPUFAs effects on immune outcomes. A systematic search was conducted using electronic databases, including OvidMedline and the Global Organization for EPA and DHA Omega-3s Clinical Study Database. Twelve studies were included in this review. EPA+DHA doses ranged from 0.6 to 4 g/d, and intervention durations ranged from 6 wk to 6 mo. Most of the studies demonstrated changes in some immune-related outcomes, including reductions in the blood markers of inflammation (interleukin-6 and C-reactive protein), a lower incidence of adverse events, and the preservation of immune cell concentrations and functions (phagocytosis and hydrogen peroxide production). However, caution is warranted due to the limited number of studies and the heterogeneity of study designs. This review discusses the limitations of current studies and the mechanistic evidence supporting the investigation and potential use of n-3 LCPUFAs during cancer chemotherapy. Future research should focus on addressing these limitations by conducting well-designed, large-scale clinical trials that clearly report the dose and duration of n-3 LCPUFAs supplementation during specific chemotherapy regimens. Despite some promising outcomes, more evidence will be needed before recommending n-3 LCPUFAs supplementation as part of chemotherapy regimens aimed at attenuating chemotherapy-induced immune alterations.

**Statement of significance**

This review provides an overview of the clinical evidence on the immunomodulatory effects of omega-3 supplementation during cancer chemotherapy. By discussing mechanisms supported by preclinical findings, this perspective review also highlights the need for higher-quality evidence to establish the potential clinical benefits of omega-3s in cancer chemotherapy.

## Introduction

Despite advances in diagnosis and treatment, cancer remains one of the leading causes of death worldwide [1]. Among cancer types, lung and bronchus (20%), colorectal (9%), pancreas (8%), and breast (7%), are projected to represent nearly 50% of the total 611,720 estimated cancer deaths in the United States in 2024 [2]. These numbers also result in high costs, exceeding US$208.9 billion, and are costly for patients, leaving many vulnerable [2]. Systemic chemotherapy is a widely used treatment consisting of a broad range of drug classes that are often combined to exert synergistic effects. Chemotherapy can be applied with curative intent, either before (neoadjuvant) or after (adjuvant) surgery, or with palliative intent (reviewed by Aboud et al. [3]). However, chemotherapy drugs lack specificity and have toxic effects on healthy tissues, limiting both the dose and frequency of treatment cycles and, ultimately, treatment efficacy. A common adverse effect is on the immune system, including leukopenia, neutropenia, and higher susceptibility to infections (reviewed by Livshits et al. [4]). Because of this, there is a growing interest in understanding the complex interaction between chemotherapy and the immune system and identifying strategies to mitigate immune adverse events.

Therapies targeting the immune system, such as the use of immune checkpoint inhibitors, adoptive T cell transfer, and cancer vaccines, have significantly transformed cancer therapy. It is now well-recognized that the immune system plays a fundamental role in the initiation, development, and eradication of tumors (extensively reviewed by Dunn et al. [5]). Growing evidence supports the fundamental role of systemic chemotherapy beyond its direct cytotoxic effects, particularly its impact on the immune system. There is a complex relationship between the immunostimulatory and immunosuppressive effects of cancer chemotherapy. For example, in breast cancer cell lines, cytotoxic drugs such 5-fluorouracil, docetaxel, and doxorubicin can exhibit immunostimulatory properties by enhancing tumor antigenicity by upregulating major histocompatibility complex-I and carcinoembryonic antigen [6,7]. These immune-enhancing properties facilitate the recognition and elimination of tumor cells by the immune system, a process known as immunogenic cell death [6,7]. However, it has been demonstrated in vitro that chemotherapy drugs, such as cyclophosphamide, can promote the depletion of T cells, impairing their proliferation and development of immune responses [8].

Systemic chemotherapy is generally considered immunosuppressive due to its low specificity for tumor cells and its direct cytotoxic effects on immune cells. Doxorubicin, a widely used anthracycline, induces “leaky gut” by damaging the intestinal epithelium and, consequently, the release of inflammatory factors into the bloodstream, contributing to systemic inflammation [9]. The induction of inflammation is shared by many chemotherapy drugs and driven by multiple mechanisms. For example, paclitaxel activates toll-like receptors [10], and similarly to other drugs, such as doxorubicin [11], cisplatin [12], and 5-fluorouracil [13], activates proinflammatory pathways, such as the nuclear factor (NF)-κB pathway, and the synthesis of inflammatory cytokines and chemokines (for example, TNF-α, IL-6, and IL-1β). Inflammation has been demonstrated to facilitate metastasis (reviewed by Karagiannis et al. [14]). Consequently, the development of adjunct therapies to enhance chemotherapy efficacy although mitigating its adverse inflammatory effects is of critical importance.

DHA and EPA are essential omega-3 long-chain PUFAs (n-3 LCPUFAs), recognized as bioactive lipid compounds with pleiotropic effects in many different types of cancer, particularly for breast [[15], [16], [17], [18]], colorectal [19], lung [20], and gastrointestinal cancers [21]. A source of DHA and EPA is fish oil supplements. There are many individual fatty acid concentrations and ratios altered by n-3 LCPUFA supplementation, including the total omega-3/omega-6 ratio in plasma and red blood cells, as well as changes in the omega-3 index (the relative percentage of n-3 LCPUFA [EPA+DHA]) in red blood cells [22]. One well-established mechanism for the anticancer effects of n-3 LCPUFAs is via changes in the fatty acid composition of cell membranes phospholipids of tumor cells. The incorporation of n-3 LCPUFAs into the plasma membrane alters different membrane properties, including fluidity, permeability, and microdomain formation. These structural modifications influence receptors, membrane-generated secondary messengers, downstream transcription factors, and oxygenated lipid-derived metabolites (reviewed by Sawyer and Field [23]). In addition to the direct effects in the membrane, n-3 LCPUFAs, after cleavage from the membrane, bind to nuclear receptors to regulate many key genes involved in cell survival and inflammation at all stages of the cell cycle (reviewed by Newell et al. [24]). Together, these alterations contribute to the activation of multiple cell death pathways, including the upregulation of intrinsic and extrinsic pathways of apoptosis [17,25] and necroptosis [18]. These cytotoxic effects mediated by n-3 LCPUFA are specific to transformed cells, conferring little to no toxicity in healthy cells and tissues, making n-3 LCPUFAs potential adjuvants without additional side effects for cancer treatment (reviewed by D’Eliseo and Velotti [26]).

There is a strong rationale for exploring the effects of n-3 LCPUFAs in attenuating immune-related side effects during cancer treatment. However, an important limitation is that the majority of studies testing the efficacy of n-3 LCPUFA supplementation have been conducted in immunocompromised animal models. Consequently, few studies have assessed immune outcomes and the tumor immune microenvironment. Clinical evidence on n-3 LCPUFAs efficacy during cancer therapy also remains limited [27]. The purpose of this study is to systematically review clinical studies and summarize the current evidence on the immunomodulatory effects of DHA and EPA supplementation in the context of cancer chemotherapy.

## Search Strategy and Tools

A literature search was conducted in the databases OvidMedline and GOED (Global Organization for EPA and DHA Omega-3s) Clinical Study Database (CSD) from September 2024 to March 2025. The GOED database is a collection of published human interventional studies using EPA and/or DHA [28]. Although the GOED CSD database is funded by the omega-3 industry, data extraction and curation were performed by GOED research assistants who are not affiliated with any industry sponsors. Briefly, 2 independent research assistants review abstracts identified through an initial PubMed search; data are then extracted from eligible studies and reviewed by another research assistant [28]. Therefore, the CSD represents an independent scientific resource, with data curated by independent researchers without conflicts of interest. The key words and subject headings were applied to identify studies to answer the following question: How does supplementation with DHA and/or EPA impact the immune alterations found in patients with cancer undergoing chemotherapy? Therefore, the keywords chosen were a combination of “patients undergoing chemotherapy” and “immune system outcomes” and “DHA or EPA.” Searches were adapted appropriately for each database. The search strategy for the OvidMedline was the combination of: “exp Neoadjuvant Therapy/ or exp Chemotherapy, Adjuvant/ or chemotherap∗.mp” AND (“immune system.mp. or exp Immune System” or “exp Lymphocytes/ or exp Immunity, Innate/ or immune function.mp. or exp Inflammation” or “blood cells.mp. or exp Blood Cells” or exp Immunity” or “exp Antibodies/ or antibodies.mp.” or “exp Febrile Neutropenia/ or neutropenia.mp.” or “infections.mp. or exp Infections/”) AND “exp Docosahexaenoic Acids/ or exp Fatty Acids, Omega-3/ or exp Eicosapentaenoic Acid/ or omega-3.mp.” The search was limited to English language and studies in humans. A total of 128 articles were initially found. Similarly, the search was conducted in the GOED CSD with the keywords: “Chemotherapy, Adjuvant,” “Chemotherapy, Curative,” “Chemotherapy, Cycles,” “Chemotherapy, Duration,” “Chemoradiotherapy,” “Chemoradiotherapy, Adjuvant,” “Neoadjuvant Therapy,” “Maintenance Chemotherapy,” “Induction Chemotherapy,” AND “Inflammation,” “Cytokines,” “Chemokines,” “Receptors, Cytokine,” “Neutropenia,” “Fever, Febrile Neutropenia,” “Infection.” A total of 33 research articles were found. Combining the results from OvidMedline and GOED CSD, a total of 161 papers were evaluated. Articles were excluded if they were duplicates, not conducted during cancer chemotherapy, or involved a combination of multiple nutrients. A total of 12 articles were included to evaluate n-3 LCPUFAs supplementation during cancer chemotherapy.

## Summary of the Clinical Evidence

The studies included in this review are summarized in Table 1 [[29], [30], [31],[32], [33], [34], [35], [36], [37], [38], [39], [40], [41]]. All the studies included in this review, except for 1, supplemented with a combination of EPA+DHA during cancer chemotherapy, and 9 of 12 studies evaluated plasma or serum markers of systemic inflammation as the main immune outcome. Three studies measured IL-6 only, 2 measured CRP only, and 4 measured both IL-6 and CRP. Three studies investigated hematological parameters, and 1 performed functional assays. Dose (0.6 – 4 g/d), duration (6 wk to 6 mo), chemotherapy regimen, and placebo composition varied greatly between studies. Of the 6 studies with a placebo, sunflower oil (*n =* 2) [29,30] was the most common, followed by olive oil (*n =* 1) [31], mineral oil (*n =* 1) [42], and corn oil (*n =* 1) [32]. Four studies lacked a placebo and used a control group that received standard care [[33], [34], [35], [36]], whereas 1 study relied on retrospective data for comparison [37], and 1 study had no control [38]. The combination of capsule count and fatty acid status represents the most reliable method for assessing compliance. Four studies used both the number of capsules returned and fatty acid status to assess compliance [31,[38], [39], [40]]. Two studies relied only on the number of capsules returned [30,32], whereas 3 assessed compliance based only on fatty acid status [29,33,34]. Three studies did not report compliance [[35], [36], [37]]. On the basis of the NIH Study Quality Assessment Tools [41] appropriate for each study design, 6 studies were classified as low risk of bias/good quality, 5 as moderate risk of bias/fair quality, and 1 as high risk of bias/poor quality (Table 1). The factors affecting study quality included the lack of blinding and placebo controls, biological measurement of compliance by fatty acid quantification, and the absence of sample size calculations (Table 1).

**TABLE 1:** Summary of the interventional studies included in this review providing n-3 capsule supplementation concomitant with chemotherapy.

Reference	Study design	Population/cancer type	Sample size (intervention; control)	Chemotherapy	Number of capsules/types of n-3 supplements	n-3 total/d; measurement of compliance	Control	Duration	Immune-related findings	Quality assessment^5^
Arsic et al. [39]	RCT, double blind	Breast cancer (IIA-IIIA)	32F (16; 16)	Anthracycline	2 omega-3 and 3 evening primrose oil capsules	1000 mg EPA +DHA + 351 mg GLA.^1^ Compliance: capsules count and fatty acids from total plasma lipids.	Placebo: mineral oil (5g/d)	12 wk	n-3: ↓ plasma IL-6. ↔ Plasma IL-8, IL-10, and hematological parameters. Placebo: **↓**leukocytes, erythrocytes, hemoglobin, platelets. **↓**Plasma IL-6	Low risk of bias.
Lustberg et al. [40]	RCT, double blind	Breast cancer (I–III)	44F (22; 22)	Anastrozol, exemestane, and letrozole	6 capsules/ triglyceride form.	430 mg EPA + 230 mg DHA in each capsule. Total of 2580 mg EPA and 1380 mg DHA. Compliance: capsules count and fatty acids from RBCs.	Placebo: mixture of fats and oils, majority palmitic acid, oleic acid, and linoleic acid.	24 wk	n-3: ↔ serum IL-6, TNFR-2, IL-17 Placebo: **↑** serum IL-17	Low risk of bias.
Mocellin et al. [33]	RCT	Colorectal cancer (II, III, and IV)	11 (6 [3M, 3 F]; 5[3M, 2 F])	Xeloda, Oxaliplatin, 5-FU and/or Leucovorin	4 capsules of fish oil (total of 2 g/d)	360 mg EPA and 240 mg DHA. Compliance: plasma fatty acids status.	Control: not supplemented	9 wk	n-3: ↓ plasma CRP and CRP/albumin ratio. ↔ Plasma TNF-α, IL-1β, IL-10, IL-17A Control: ↑CRP	Moderate risk of bias. Lack of blinding and no sample size calculation.
Chagas et al. [34]	RCT	Hematological	22 (9 [5F, 4M]; 13 [5F, 8M])	Type not specified	2 capsules of fish oil (total of 2g/d).	610 mg n-3 PUFAs (367 mg EPA and 243 mg DHA). Compliance: plasma fatty acid status.	Control: not supplemented	9 wk	n-3: ↔ Serum CRP and CRP/albumin. Control: ↑ RBCs, hemoglobin, hematocrit, leucocytes	Moderate risk of bias. Lack of blinding and no sample size calculation.
Finocchiaro et al. [31]	Multicenter, RCT double-blind trial	Advanced NSCLC	27 (13 [8M, 5F]; 14 [11M, 3F])	Gemcitabine and cisplatin	4 capsules (source unknown)	510 mg EPA and 340 mg DHA. Compliance: capsule count and fatty acids from plasma and RBCs total lipids.	Placebo: 850 mg olive oil	66 d (∼9 wk)	n-3: ↓ plasma IL-6; Placebo: ↑CRP and IL-6	Low risk of bias. No sample size calculation.
Lu et al. [37]	Retrospective cohort study	Advanced NSCLC (IIIA and IIIB)	137 (77 [43M, 34F]; 60 [33M, 27F])	Cisplatin, docetaxel, bevacizumab	Not specified	510 mg EPA and 200 mg DHA Compliance: not reported.	Control: not supplemented	6 wk	n-3: ↓ plasma CRP and IL-6. ↔ TNF-α or PGE2	High risk of bias. Retrospective study, no compliance accessed, no control for confounding factors.
Silva et al. [35]	RCT	Colorectal cancer	23 (11 [3F, 8M], 12 [3F, 9M])	Type not specified	4 capsules of fish oil (total of 2g/d)	600 mg EPA+DHA. Compliance: not reported.	Control: not supplemented	9 wk	n-3: ↓ serum CRP/albumin. ↔ Serum IL-6, TNF- α, IL-1β	Moderate risk of bias. Lack of blinding and placebo, and no sample size calculation.
Hutchins-Wiese et al. [29]	RCT, double blind	Breast cancer	38F (20; 18)	Aromatase inhibitors	7 capsules of fish oil	4 g EPA+DHA (2520 mg EPA +1680 mg DHA)^2^. Compliance: serum fatty acids.	Placebo: sunflower oil (9% linoleic acid, 83% oleic acid)	3 mo	↔ Serum IL-6, IL-1β, hs-CRP, MIF, TNF-α.	Low risk of bias. No sample size calculation (pilot study).
Haidari et al. [32]	RCT, double blind	Colorectal cancer (II and III)	80^3^ (20 [12F, 8M], 20 [8F, 12]	Type not specified	2 capsules of fish oil	660 mg omega 3 (118 mg EPA + 500 mg DHA). Compliance: capsule count.	Placebo: corn oil	8 wk	n-3: ↓ serum TNF-α. ↔ Serum NF-kB, IL-1β, IL-6, IL-8, CEA	Low risk of bias. No fatty acid measurement (compliance).
de la Rosa Oliva et al. [30]	RCT, double blind	Breast cancer (IIA–IIIB)	52F (26; 26)	Adriamycin, cyclophosphamide, paclitaxel, +/- trastuzumab	4 fish oil capsules (total of 2.4 g)	1.6 g EPA and 0.8 g DHA. Compliance: capsule count.	Placebo: sunflower oil	6 mo	n-3: ↔ Hematological parameters	Low risk of bias. No sample size calculation and no fatty acid measurement (compliance).
Bougnoux et al. [38]	Prospective open-labeled phase II trial	Breast cancer metastatic	25F	FEC: fluorouracil, epirubicin, cyclophosphamide	0.5 g capsules of DHA-enriched triglyceride oil of algal origin	1.8 g DHA. Compliance: capsule count and plasma fatty acids.	None	18 wk	Higher n-3: ↓ incidence of thrombocytopenia and anemia. ↔ Neutropenia.	Moderate risk of bias. Lack of blinding and lack of placebo.
Bonatto et al. [36]	RCT	Various, majority gastrointestinal	38 (19 [12M, 7F]; 19 [10M, 9F])	5-fluorouracil and leucovorin	Unknown/ total of 2g/d of fish oil	0.3 g EPA and 0.4 g DHA. Compliance: not reported.^4^	Control: not supplemented	8 wk	Control: ↓ PMNC number and function (phagocytosys and hydrogen peroxide production)	Moderate risk of bias. Lack of blinding, lack of placebo, and no sample size calculation.

Abbreviations: CEA, carcinoembryonic antigen; F, female; FACT-ES, Functional Assessment of Cancer Therapy-Endocrine Subscale; GLA, gamma-linolenic acid GGT, hs-CRP, high-sensitivity c-reactive protein; M, male; NSCLC, non-small cell lung cancer; PMNC, polymorphonuclear cell; RBCs, red blood cells; RCT, randomized controlled trial.

1 N-3 capsules contained the n-6 polyunsaturated fatty acid gamma-linolenic acid.

2 All participants received calcium carbonate (1000 mg/d) and cholecalciferol (800 IU/d).

3 Two additional groups: one with vitamin D (50,000 IU weekly, *n* = 21) alone and other with omega 3 + vitamin D (*n =* 20).

4 Compliace was not assessed by fatty acid status or capsule counts. However, the study evaluated the fatty acid composition of PMNCs.

5 Study quality assessment via NIH Study Quality Assessment Tools [42] as appropriate for each study design.

## Impact on Patients with Breast Cancer

Inflammation is recognized as one of the hallmarks of cancer [43]. Chronic inflammation is characterized by the persistence of inflammatory factors, which may lead to tissue damage and contribute to cancer progression [44]. The proinflammatory cytokine IL-6, primarily produced by macrophages, induces the activation of transcription factors in hepatocytes and other immune cells, including the signal transducer and activator of transcription 3 and NF-kB [45,46]. The activation of these pathways stimulates the synthesis of C-reactive protein (CRP) by hepatocytes in the liver, and it is recognized as one of the main acute-phase proteins [45,46]. Elevation of IL-6 and CRP are well-recognized features of cancer progression and considered predictors of poor prognosis and survival, and are found to be associated with chemotherapy resistance in breast [47], colorectal [48], lung [49], and gastrointestinal cancers [50,51]. The CRP/albumin ratio is considered another important marker, as physiological stress leads to an elevation in CRP concentration and, consequently, a downregulation of albumin synthesis [52]. Therefore, a higher CRP/albumin ratio is a biomarker of inflammation and is considered a prognostic factor for overall survival in both gynecological [53] and colorectal cancers [54].

The plasma or serum concentrations of IL-6 and CRP were evaluated in 9 of 12 interventional studies (Table 1). Compared with a placebo, n-3 LCPUFA supplementation reduced plasma concentrations of IL-6 in breast (*P <* 0.001) [39] and lung cancers (*P <* 0.05) [31,37]. Additionally, supplementation reduced (*P <* 0.05) CRP concentrations in lung [31,37] and colorectal cancers [33]. Systemic inflammatory markers were evaluated in patients with breast cancer (stages I–III) supplemented with a combination of 1000 mg EPA+DHA and 351 mg gamma-linolenic acid (GLA) (*n =* 14) during anthracycline chemotherapy (12 wk). At the end of treatment, plasma IL-6 concentrations were lower in the LCPUFA group than in the mineral oil placebo group [median (range): 9.4 (0.95–10.54) compared with 12.8 (0.9–40.1) ng/mL, *P <* 0.001] [39]. However, in this study, the LCPUFA treatment did not significantly change plasma concentrations of IL-10, IL-8, and TNF-⍺. The concentration of IL-6 was lower (*P <* 0.001) in both groups at the end of chemotherapy, which is counter to the well-documented proinflammatory effects of anthracyclines. Anthracyclines, such as doxorubicin, are known to induce oxidative stress and inflammation, typically leading to elevated systemic IL-6 and TNF-⍺ [11]. Although the study by Arsic et al. [39] used changes in the inflammatory markers as the primary outcome, there was no explanation of sample size calculation, which could have influenced the lack of changes in systemic markers.

Changes in proinflammatory cytokines during chemotherapy treatment were evaluated in 2 other randomized controlled trials (RCTs) in patients with breast cancer. Patients (*n =* 22, stages II–III) were randomly assigned to receive 1.38 g of DHA and 2.58 g of EPA during 24 wk of treatment with a combination of anastrozole, exemestane, and letrozole [40]. Changes over time were compared with a placebo composed of a mixture of oils (oleic, linoleic, and palmitic acid). No significant changes in the serum concentration of IL-6, TNFR-2, or IL-17 were found. Similarly, no differences were found in the concentrations of serum IL-6, IL-1β, macrophage migration inhibitory factor (MIF), and TNF-⍺ in patients with breast cancer (*n =* 20) supplemented with 1.68 g of DHA and 2.52 g of EPA daily during 3 mo of aromatase inhibitors [29]. However, there was an increase in high sensitive (hs)-CRP in the n-3 group at the end of the study (2.86 ± 3.29 compared with 4.79 ± 6.59 mg/L, *P =* 0.027) and a greater increase than in the control group (7.78 ± 8.38 compared with 2.38 ± 3.25 mg/L, *P =* 0.022) [29]. This is the only study that reported an increase in proinflammatory markers during cancer chemotherapy although supplementing with n-3 LCPUFAs.

Patients with stages IIA–IIIB breast cancer undergoing neoadjuvant chemotherapy demonstrated no differences in the incidence of adverse events, including leukopenia, neutropenia, and anemia between the fish oil supplemented group (2.4 g/d of fish oil, DHA/EPA not specified) and the placebo group (sunflower oil) [30]. This was evaluated after 6 mo of combined chemotherapy with adriamycin, cyclophosphamide, paclitaxel, and with or without trastuzumab [30]. Similarly, 2 other studies, 1 involving patients with breast cancer and another involving patients with hematological malignancies, reported no differences in changes in the concentrations of leukocytes, erythrocytes, hemoglobin, or platelets compared with the controls [34,39]. Consistent with these 2 studies, Murphy et al. [55] found no differences in the incidence of grades 3 or 4 toxicities, including neutropenia, nausea, and vomiting, between patients with non-small cell lung carcinoma (NSCLC) supplemented with 2.5 g EPA+DHA/d during ∼10 wk of palliative or adjuvant chemotherapy [56]. Interestingly, Arsic et al. [39] reported a decrease in leukocyte and erythrocytes concentration in both the placebo and n-3 LCPUFAs groups at the end of anthracycline chemotherapy (*P <* 0.05), whereas Chagas et al. [34] reported decreases in these 2 cell types only in the control group (*P <* 0.05). Contradictory to these findings, an open-label trial found that at the end of supplementation with 1.8 g DHA (algae-enriched triglycerides) for metastatic breast cancer (18 wk), the incidence of anemia (*P =* 0.01) and thrombocytopenia (*P =* 0.02) were less frequent when DHA was highly incorporated (>2.5%) in plasma phospholipids [38]. To date, no placebo-controlled trials support the protective effects of n-3 LCPUFAs against chemotherapy-induced hematological toxicity.

## Colorectal Cancer

Changes in inflammatory markers after n-3 LCPUFA supplementation have also been reported in patients with colorectal cancer. A systematic review concluded that there was sufficient evidence for n-3 LCPUFAs to reduce systemic CRP concentrations in patients with colorectal cancer, suggesting their potential anti-inflammatory benefits in this population [57]. Consistent with this, a study on patients with colorectal cancer (*n =* 24) with stages II and III found that supplementation with 500 mg DHA and 118 mg EPA daily for 8 wk reduced the concentration of serum TNF-⍺ compared with placebo (corn oil) ( −0.61 ± 0.68 compared with −0.08 ± 0.65 pg/mL, *P =* 0.01) [32]. However, no significant differences between groups were found for IL-1β, IL-6, and IL-8 [32].

Consistent with these findings, another study in patients with colorectal cancer investigated the effects of fish oil supplementation (2 g/d, providing 360 mg EPA and 240 mg DHA; *n =* 6) for 9 wk while undergoing chemotherapy (combination of xeloda, oxaliplatin, 5-fluorouracil, and leucovorin) [33]. There were no significant changes in plasma concentrations of TNF-⍺, IL-1β, IL-10, and IL-17A. However, compared with baseline, plasma CRP concentration and the CRP/albumin ratio increased (*P <* 0.05) in the control group at the end of therapy, whereas these 2 biomarkers decreased (*P <* 0.05) in the fish oil-supplemented group. This resulted in differences in CRP and the CRP/albumin ratio between the 2 groups (*P <* 0.05) [33]. Similarly, another study on patients with colorectal cancer (stages I–IV) investigated supplementation with 2 g/d of fish oil (*n =* 10, providing 600 mg DHA + EPA) for 9 wk during chemotherapy (regimen unknown) [35]. The study found a trend toward lower CRP concentration (*P =* 0.06) and a reduced CRP/albumin ratio in the fish oil-supplemented group (*P =* 0.09), without significant changes in systemic inflammatory markers (IL-6, IL-1β, and TNF-⍺). [35].

## Leukemia and Lymphoma

A single study evaluated the effect of n-3 LCPUFAs in mixed hematological (leukemia and lymphoma) malignancies, and 2 other studies examined the effect in lung cancer. Supplementation with fish oil (2 g/d, 367 mg EPA + 243 mg DHA) for 9 wk during chemotherapy reduced CRP concentration (*P =* 0.04) [34]. However, changes in CRP concentration (*P >* 0.05) and CRP/albumin ratio (*P* value not provided) were not different between the treatment and control groups [34]. In contrast, patients with advanced NSCLC (*n =* 77), receiving cisplatin-based chemotherapy, were supplemented with 510 mg EPA + 200 mg DHA for 6 wk [37]. Compared with baseline, plasma concentration of IL-6 and CRP were decreased in the n-3 LCPUFA group but were increased in the control group, resulting in significant differences between the 2 groups (*P* < 0.01 for both) [37]. This study did not find differences in the changes in TNF-⍺ or prostaglandin E2 concentrations between groups [37]. Similar findings were observed with patients with lung cancer (*n =* 13) supplemented with 510 mg of EPA+ 340 mg DHA for 9 wk, which resulted in lower plasma concentrations of IL-6 and CRP (*P* < 0.05 for both) after 9 wk of treatment, compared with the placebo group (850 mg olive oil) [31].

## Immune Cell Function

The effects of n-3 LCPUFA supplementation on immune cell function during cancer therapy were evaluated in only 1 study. Bonatto et al. [36] found a significant effect on polymorphonuclear cell (PMNC) function after 8 wk of fish oil supplementation (2 g/d, 0.3 g EPA+ 0.4 g DHA) during adjuvant chemotherapy (8 wk, 5-flourouracil and leucovorin) for gastrointestinal cancer. Compared with the fish oil group, participants in the non-supplemented group had a reduction in PMNC concentration (2.13 ± 0.05 compared with −2.69 ± 0.03 × 10^6^/mL, *P <* 0.05), phagocytosis (*P <* 0.05), and hydrogen peroxide production (*P <* 0.05). However, the study did not evaluate cytokine or other inflammatory markers in participants’ blood.

In summary, there is limited evidence on the effects of n-3 LCPUFAs supplementation on systemic inflammatory markers and most findings are supported by a single study. Although no differences were observed for other pro- (IL-1β, MIF, TNF-α, IL-8, IL-17A) and anti-inflammatory (IL-10) markers, studies suggest that n-3 supplementation reduces CRP levels in colorectal cancer and CRP and IL-6 in lung cancer. However, only the study by Finocchiaro et al. [31] included a placebo group. The other 2 studies compared their results with a non-supplemented group [33,37]. One study reported that n-3 LCPUFAs can maintain PMNC number and function during chemotherapy [36]. Few studies have examined the effects of n-3 LCPUFAs on chemotherapy-induced hematological toxicity, but 1 study [38] found that n-3 LCPUFAs attenuated this toxicity. Despite the well-documented dynamic changes in CRP and other inflammatory immune markers, only 3 [30,31,40] of 12 studies assessed systemic markers throughout the course of treatment.

## Discussion

**FIGURE 1:**
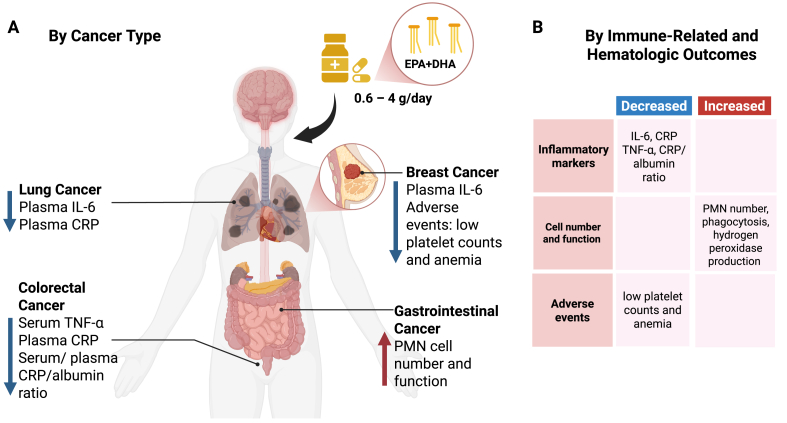
Summary of the current evidence from studies on the beneficial effects of n-3 long-chain PUFAs supplementation on immune and hematologic outcomes during cancer chemotherapy. The data for this figure are derived from the studies described in Table 1. (A) Findings summarized by cancer type. (B) Findings summarized by immune-related and hematologic outcomes. CRP, C-reactive protein; PMN, polymorphonuclear. Created in BioRender. Munhoz, J. (2025) https://BioRender.com/59hmnq9

The current evidence of the effects of n-3 PUFAs supplementation on the immune system of participants undergoing cancer chemotherapy are summarized in Figure 1. Most of these findings represented in Table 1 are supported by single studies and, therefore, they should be interpreted with caution. Beneficial effects on blood markers of systemic inflammation (plasma IL-6 and serum CRP) were the most common reported outcomes. However, the majority of immune outcomes assessed in the trials included in this review were limited to these 2 inflammatory markers, leaving many other aspects of immune function unexplored.

N-3 LCPUFAs exert immunomodulatory properties through multiple pathways, many of which are initiated or associated with the cellular membrane (reviewed by Calder [58]). One key immune mechanism involves the oxidation of PUFAs in the plasma membrane, which results in the formation of lipid mediators that regulate inflammation and immune function [59]. Among these products, specialized pro-resolving mediators, derived from n-3 PUFA oxidation, are crucial in resolving non-infectious inflammation, primarily by activating G-protein-coupled receptors on myeloid lineage cells [60]. Macrophages acquire a pro-resolving phenotype that it has been shown to impair tumor progression and modify the tumor microenvironment [61,62]. In addition to n-3 LCPUFAs' effects on lipid mediators, these fatty acids modulate immune cell function via direct interactions with surface and intracellular receptors and by modulating membrane protein function through alterations in membrane fluidity and lipid raft formation [58]. Our group previously demonstrated in a patient-derived xenograft model of triple-negative breast cancer that DHA downregulates the gene expression of NF-κB and TNFR1 in tumor cells [18]. Although this study focused on the direct effects within the tumor, the reduction of these inflammatory genes may be one of the mechanisms for the reported decrease in systemic inflammatory markers when patients were supplemented with n-3 LCPUFAs.

N-3 LCPUFAs have been shown to modulate immune cell function in rodent models. For example, in a colorectal cancer model, fish oil supplementation attenuated chemotherapy-induced lymphocyte depletion although preventing excessive neutrophil accumulation, normalizing the neutrophil-to-lymphocyte ratio (NLR) [63]. NLR has emerged as an important prognostic factor in cancer treatment, with a lower ratio being associated with improved survival for colorectal [64] and breast [65] cancers. Neutropenia is a common immune-related adverse event of chemotherapy, and during cancer progression, neutrophils can acquire an immunosuppressive phenotype under the influence of tumor-derived factors. This phenotype contributes to disease progression by releasing cytokines and other regulatory molecules [66]. Bonatto et al. [36] demonstrated that n-3 LCPUFA supplementation increased cell number and enhanced immune function in isolated PMNCs by improving phagocytosis. Although the exact mechanism was not further explored in this study, these results suggest that n-3 LCPUFAs may help preserve immune function during chemotherapy treatment. Other rodent studies have demonstrated that n-3 LCPUFAs influence NK [67] and B cells [68] functions, both of which play crucial roles in antitumor immunity and are often suppressed during chemotherapy [69]. Future studies should consider investigating these cells and their function.

Some studies have provided n-3 LCPUFAs fatty acids with other nutrients with immune benefits, such as vitamins A and D, ribonucleic acids, arginine, and glutamine. This form of nutrition therapy has been used to treat head and neck cancers where food intake is severely compromised. Despite some promising findings [70], a recent systematic review (*n =* 7 studies) concluded that the overall level of evidence for the protective effects of treating patients with immunonutrients and mixtures of these against immune side effects during cancer chemotherapy remains inconclusive [71]. The inconclusive findings can be attributed to factors similar to those identified in our review: study heterogeneity, inconsistency in reporting, and lack of comprehensive assessments of immune alterations [71].

Cancer therapy is often associated with reduced food intake, weight loss, and significant impacts on nutritional status. Variations in weight and dietary intake are important confounding factors that need to be considered. Few of the studies estimated or measured dietary intake during treatment, nor did they monitor fatty acid status beyond a single timepoint. There are mixed findings of the effects of n-3 LCPUFAs supplementation in cancer cachexia, a multifactorial syndrome characterized by weight loss, loss of skeletal muscle mass, and altered metabolism [72]. A recent meta-analysis of 7 RCTs, including studies with participants with pancreatic, lung, and head and neck cancers, concluded that n-3 LCPUFAs supplementation (ranging from 2 to 4 g/d) increased body weight [73]. This effect followed a nonlinear dose relationship and was only present in a subgroup analysis of participants aged 67 or older with a baseline body weight of 60 kg or less [73]. In contrast, another systematic review conducted with studies of patients with cancer cachexia found no effects of n-3 supplementation on inflammatory markers such as CRP or IL-6 [74]. The limited assessment of body weight across studies restricted interpretation. Although a proinflammatory state is known to contribute to protein catabolism and further exacerbate cachexia [72], the effects of n-3 LCPUFAs in this context remain unclear.

A strength of the assessed studies is that most verified compliance through fatty acid status analysis and used placebos and doses unlikely to significantly impact overall dietary intake. However, the lack of repeated fatty acid status and dietary intake assessments remains a potential confounding factor. Because chemotherapy is typically administered in repeated cycles throughout treatment, it presents multiple opportunities for assessment to capture the potential alterations in immune function and dietary intake during therapy. One critical factor in this context is the dose–response relationship by which inflammatory immune cells incorporate n-3 LCPUFAs, a process that is both dose- and time-dependent until saturation is reached [75,76]. On the basis of clinical evidence, some reviews suggest that the most effective dosage of EPA+DHA for cancer chemotherapy is ≥ 2 g/d [77,78]. Although half of the studies included in this review used doses above 2 g/d, more recent findings indicate that even lower doses may have a biological effect. For example, a recent open-label trial found that compared with a control group, daily supplementation with 360 mg EPA and 240 mg DHA to patients with breast cancer resulted in lower concentrations of hs-CRP at the end of 9 wk of chemotherapy treatment [79]. Because none of the studies in this review assessed a dose–response relationship, and no clear association between higher doses and better effects was established, the optimal dosage and its impact on immune outcomes in cancer chemotherapy remain unclear.

A growing body of literature, including both in vivo and in vitro studies, supports the distinct effects of DHA and EPA on the tumor [80] and the immune system [81,82]. However, there were insufficient studies published to enable a comparison of the type of n-3 LCPUFA supplements used or evaluate the individual and combined effects of DHA and EPA on immune outcomes. Most studies were powered on clinical outcomes other than immune function or biomarkers, limiting definitive conclusions. This limitation highlights a critical gap in the current literature, as findings and mechanisms supported by preclinical studies have yet to be translated to humans.

## Perspectives

**FIGURE 2:**
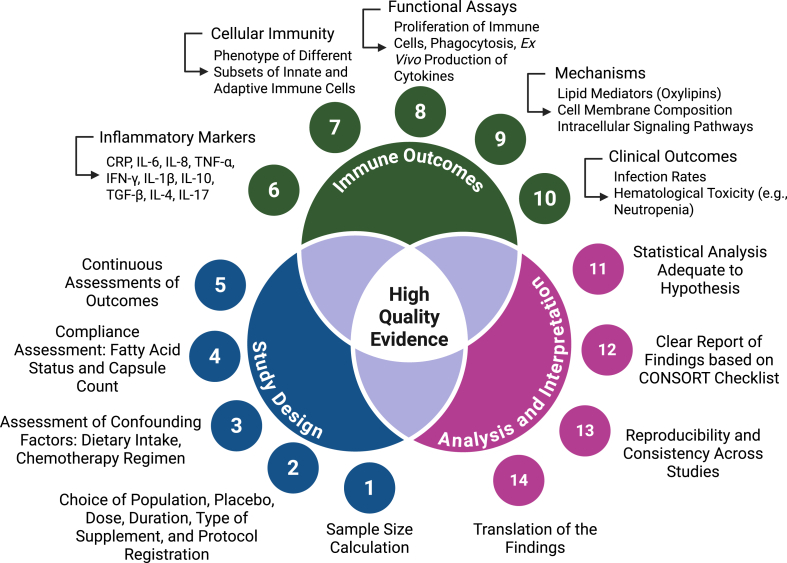
Proposed model of study components for higher-quality evidence. The initial steps involve the study design and include sample size calculation, definition of the study protocol based on existing literature, assessment of confounding factors and compliance, and evaluation of multiple timepoints. Ideally, studies aimed at evaluating immune outcomes should expand their methods to include inflammatory markers, cellular immunity, and functional assays, and integrate these with mechanisms and clinical outcomes. Lastly, studies should report findings based on robust statistical analyses and follow established guidelines (for example, CONSORT checklist) for clear and transparent reporting. Together, these steps have the potential to enhance reproducibility and consistency across studies and facilitate the translation of findings into treatment recommendations. IFN-γ, interferon gamma; TGF-β, transforming growth factor beta. Created in BioRender. Munhoz, J. (2025) https://BioRender.com/z47qrdk

The studies included in this review suggest that there is heterogeneity in immune alterations that occur with different chemotherapy regimens. To date, there is no consensus on the optimal dose, duration, type of supplement, or which patients would benefit most from supplementation. Figure 1 summarizes the available evidence (found in Table 1). Larger, adequately powered studies with well-defined supplements and clinically relevant immune outcomes are needed to expand current knowledge and elucidate the effectiveness of n-3 PUFA supplementation during chemotherapy treatment. We suggest that future studies consider our proposed model of study components so as to obtain higher-quality evidence (Figure 2). On the basis of preclinical studies, future trials could provide the necessary evidence to support the inclusion of n-3 PUFA supplementation during chemotherapy treatment.

## Author contributions

The authors’ responsibilities were as follows – JM, CJF: designed the review and selection of studies; JM: wrote the initial draft of the manuscript; VM, CJF: reviewed and edited the drafts; and all authors: contributed to the final content and read and approved the final version of the manuscript.

## Funding

This research was funded by the Canadian Institutes of Health Research (grant number: RES0037745). CJF holds a Canadian Research Chair Tier 1 in Human Nutrition and Metabolism. JM received scholarships from the Cancer Research Institute of Northern Alberta (CRINA), Alberta Graduate Excellence, Hazel McIntyre Summer Research Award, Dr. Elizabeth A Donald MSc, and Anthony Fellowship in Human Nutrition from the Department of Agricultural, Food & Nutritional Science (University of Alberta, Edmonton, Alberta).

## Conflict of interest

CJF is an Editorial Board Member of the Advances in Nutrition and played no role in the Journal’s evaluation of the manuscript. All other authors report no conflicts of interest.
